# Immediate coiling of a gastroduodenal arterial bleeding in a case of haemorrhagic shock without haematemesis, a case report

**DOI:** 10.1016/j.amsu.2022.104146

**Published:** 2022-07-12

**Authors:** Donald Schweitzer, Sanne W. De Boer, Roel M.M. Bogie, Daniel Keszthelyi, Dave H. Schweitzer, Stefan A.W. Bouwense

**Affiliations:** a Department of Surgery, Maastricht University Medical Centre, Maastricht, the Netherlands; b Department of Radiology, Maastricht University Medical Centre, Maastricht, the Netherlands; c Department of Internal Medicine, Division of Gastroenterology and Hepatology, Maastricht University Medical Centre, Maastricht, the Netherlands; d Department of Internal Medicine, Reinier de Graaf Hospital, Delft, the Netherlands

**Keywords:** Coiling, Computed tomography angiography, Diverticular bleeding, Digital subtraction angiography, Surgical emergency, Upper GI

## Abstract

**Introduction:**

and importance: Upper gastrointestinal (GI) bleeding is common in the clinic. In combination with haemorrhagic shock, morbidity is high. Rapid diagnosis and treatment can save lives. With the introduction of precision imaging several treatment options are feasible. Up-to-date diagnosis and treatment requires expertise from interventional radiology, gastroenterology and surgery to form a dedicated intervention team. This is illustrated by a typical case.

**Case presentation:**

We report a 78-year-old otherwise healthy male with a severe diverticulum bleeding. He was initially diagnosed with acute pancreatitis. Approximately 60 minutes after CT scanning, he became haemodynamically instable. He also vomited coffee-like fluid but no clear blood or clots. A repeated CT scan showed active bleeding in the retroperitoneal space highly suspicious for a diverticular bleeding just outside the lumen of the duodenum. An acute multidisciplinary intervention team immediately decided not to perform endoscopy (according to the upper GI bleeding guidelines) but to extend the imaging procedure with digital subtraction angiography (DSA). By this time, active bleeding from a side branch of the gastroduodenal artery was noted and successfully coiled.

**Clinical discussion:**

Guidelines determine day-to-day management in clinical medicine. Still, there is an exception to every rule. The case presented here was typical of upper GI bleeding with haemodynamic instability and signs of shock, but without haematemesis. This combination indicated a bleeding from somewhere outside the lumen of the GI tract. Instead of endoscopy, the acute intervention team decided to perform CT angiography (CTa) with subsequent DSA. On imaging, the bleeding focus was immediately identified and treated by coiling.

**Conclusion:**

Performance of CTa immediately followed by DSA and no endoscopy was decided by an acute intervention team in a patient with upper GI bleeding and haemorrhagic shock. Swift coiling of the bleeding artery outside the GI tract lumen was successful. The team in charge relied on a hybrid multifunctional unit fully equipped to perform interventional radiologic as well as GI procedures.

## Highlights

• Direct computed tomography angiography could be an option in case the patient vomits no pure blood.
• Multidisciplinary revaluation is essential in an unstable patient with an upper GI bleeding until the situation is stabilized.
• The role of coiling is growing, with this important technic a laparotomy could be avoid.

## 1 Introduction

Upper gastrointestinal (GI) bleedings are frequently seen, the incidence variates per country and also the reason for the bleeding [1]. The incidence of spontaneous duodenal diverticular bleedings is estimated to be 0,14% [2]. The American College of Gastroenterology recommends immediate endoscopy in patients with Upper GI bleeding. Endoscopic re-examination after endoluminal intervention remains the cornerstone in follow-up [3,4]. Dutch guidelines agree with this approach [5]. We here report a patient in haemodynamic shock due to an arterial bleeding outside GI lumen. Diverticular arterial bleeding was diagnosed and treated. This case report has been reported according to the SCARE criteria 2020 [6].

## 2 Case presentation

**Fig. 1:**
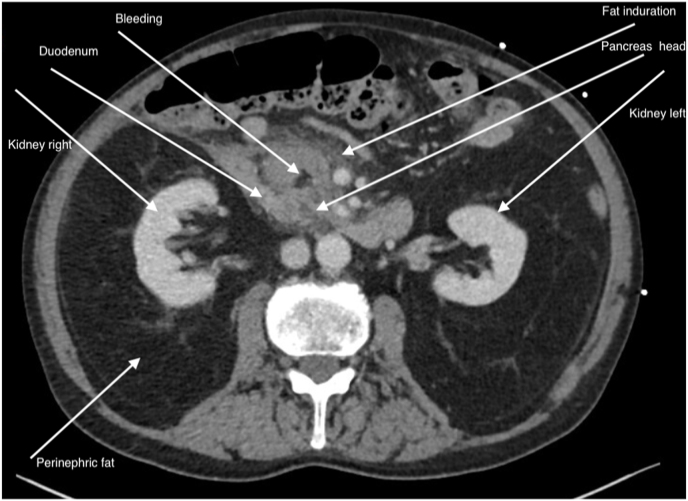
Transversal CT image: a bit fluid around the head of the pancreas.

A 78-year-old, otherwise healthy, non-smoking, and non-alcoholic white male (BMI 21.6 kg/m2) suddenly complained of upper abdominal pain for some 14 hours. He had a radical prostatectomy for prostate cancer, his further medical history was unremarkable, he was on no chronic medication. The patient once vomited a 'coffee-like substance' at home. Stools were normal. When admitted to the Emergency Department of the Academic Medical Hospital, Maastricht, The Netherlands, he was initially not in shock. At presentation, the patient had signs of a paralytic ileus without any suspicion of peritonitis. Laboratory (Lab.) results were all within Lab. ref. Range but haemoglobin was decreased (6.9 mmol/L) and Lipase was increased (3854 U/L). CT scan (portal venous phase) showed fluid around the head of the pancreas (Fig. 1). Therefore, the patient was initially diagnosed with pancreatitis. Hemodynamic shock developed in 60 minutes with a second vomiting of 'coffee-like substance'.

## 3 Investigation

**Fig. 2:**
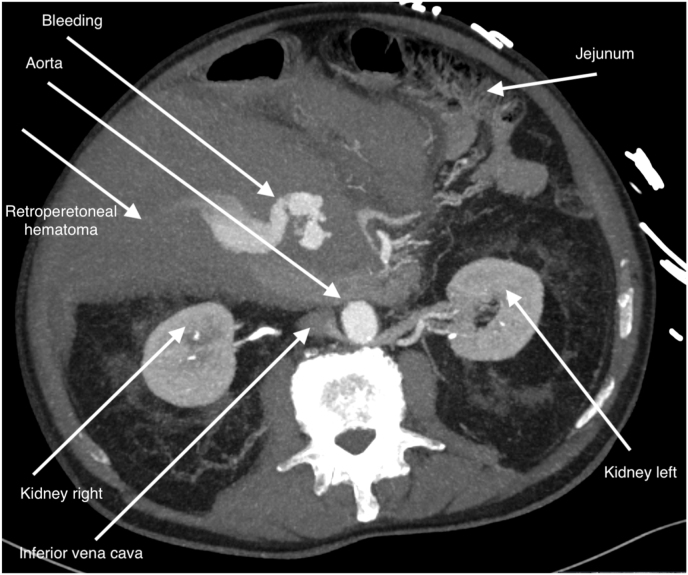
Transversal CT image: in a short period, a peritoneal hematoma develop with active leakage.

**Fig. 3:**
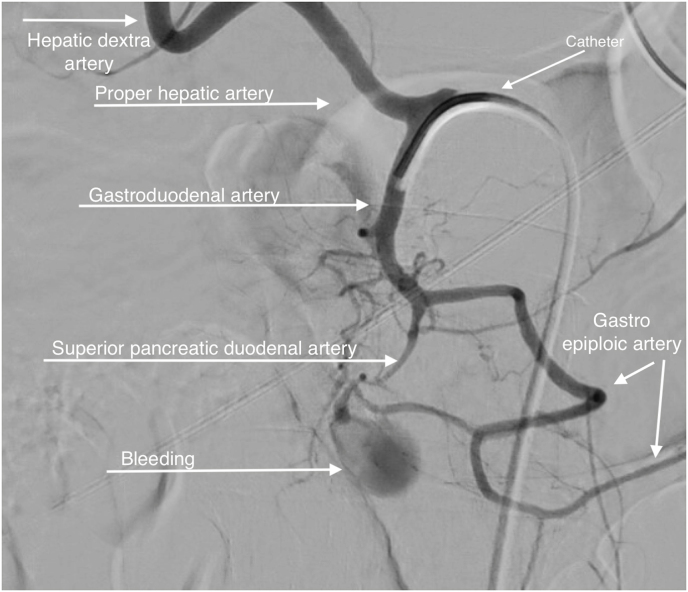
Digital subtraction angiography: before coiling, the leakage shows where the contrast enters the retroperitoneal space.

When the patient became haemodynamically unstable, CT angiography (CTa) was performed followed by digital subtraction angiography (DSA). The results showed an extravasate originating from a side branch of the gastroduodenal artery adjacent to a duodenal diverticulum (Fig. 2, Fig. 3 and video 1). Follow-up Lab. showed a Haemoglobin of 4.5 mmol/L.

Supplementary video related to this article can be found at https://doi.org/10.1016/j.amsu.2022.104146

The following is the supplementary data related to this article:

Video 1 Digital subtraction angiography: before coiling, the leakage is around the diverticulum. The contrast shows where the contrast enters the retroperitoneal space. Video 1

## 4 Differential diagnosis

Spot diagnosis from CTa was a bleeding from a duodenal diverticulum. Still, some other causes were considered like bleeding from a local aneurysm or malignancy or gastric and/or duodenal laesion (i.e. malignancy of the stomach or gastric or duodenal ulcer).

## 5 Treatment

**Fig. 4:**
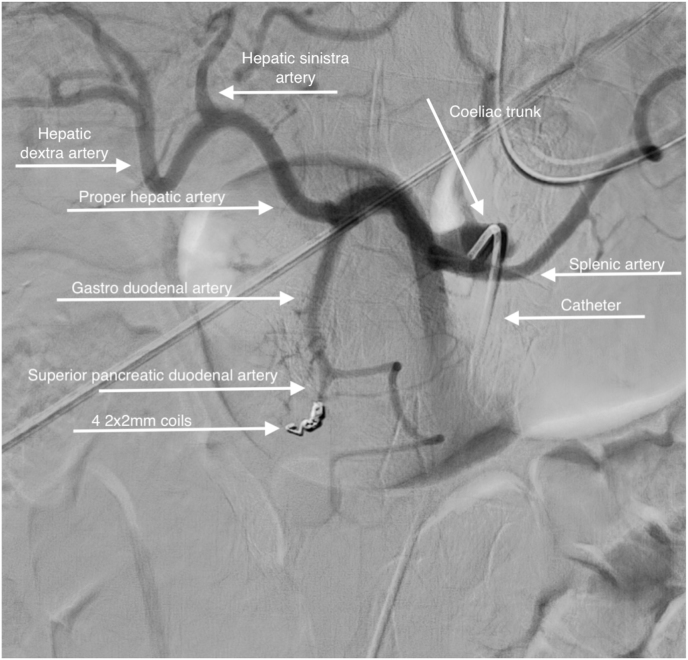
Digital subtraction angiography: the coils are placed preventing further leakage to the retroperitoneal space.

A DSA was performed after careful but prompt decision making by the members of the acute intervention team. A catheter was inserted into the superficial femoral artery under local anaesthetic. A catheter inserted in the (under local anaesthetic) and advanced into the gastroduodenal. An active blush was noticed at a side branch of the gastroduodenal artery (Fig. 3 + video 1). Effective coiling was performed using four 2 × 2 mm coils. This resulted in a complete disappearance of the blush. (Fig. 4 + video 2). There were no other leakages. Because of haemodynamic instability at presentation, follow-up observation was initially at the Intensive Care Unit (ICU). The patient made a full recovery and was discharged at day 6.

Supplementary video related to this article can be found at https://doi.org/10.1016/j.amsu.2022.104146

The following is the supplementary data related to this article:

Video 2 Digital subtraction angiography: the coils are placed preventing further leakage to the retroperitoneal space. Video 2

## 6 Outcome and follow-up

It took 6 days of hospitalization for complete recovery: 2 days in the ICU and 4 days in the surgical ward. A follow-up CTa at day 2 revealed no recurrent bleeding, the coils were still in the correct position and some residual blood was observed in the retroperitoneal cavity (i.e., around the head of the pancreas). Several checks after coiling were unremarkable; lipase levels normalized. A follow-up CTa was made 8 weeks after hospital discharge showing residual hematoma around the head of the pancreas, no other abnormalities. Endoscopy was performed 6 months later, which showed grade A reflux oesophagitis but no macroscopic intraluminal abnormalities.

## 7 Discussion

Haemorrhagic shock caused by bleeding from the gastroduodenal artery is rarely reported. The incidence of it is unknown. Most cases of upper GI bleeding are caused by gastric or duodenal ulcers or malignancy [1]. Patients in haemorrhagic shock have usually clinical signs of active bleeding like haematemesis or melaena. Vomiting coffee-coloured stomach contents is quite rare in upper GI bleeding patients who are in shock. His rapid deterioration prompted us to rush him to a multi-purpose unit in the emergency department. All members of the acute intervention team were consulted on site. Based on the agreement of all three members, it was agreed not to perform emergency endoscopy but to proceed with a CTa [7]. This decision was completely different from the recommendations in the upper GI bleeding guideline [4,8,9]. Note that in the case of upper GI bleeding with production of whole blood or whole blood clots, rescue endoscopy should always be 'step number one' [[3], [4], [5]]. In case of a negative endoscopy, CTa provides crucial information about possible bleeding loci outside the intestinal lumen [10,11]. A CTa also serves as a roadmap to make the decision to perform a DSA for careful bleeding localization and intravascular intervention [[11], [12], [13], [14], [15]] and (Fig. 1, Fig. 2, Fig. 3, Fig. 4).

Post-mortem studies have shown that 22% of all GI diverticula are located in the duodenal region [15,16]. Upper GI bleeding from diverticula is estimated to account for 0.14% of all cases of GI bleeding [2]. The role of endoscopy remains controversial. To our knowledge, the percentage of false negative endoscopies is unknown [2,17,18].

Diagnostic performance of CTa with DSA continues to improve. In case of this patient, we used a Siemens Somatom Definition Flash CT scanner (5 mm slices). New generation scanners up to 2 mm slices may perform even better with very small volume bleedings of 0.1–0.25 ml/h [7,8,17,19].

To our knowledge, there are no detailed reports of patients with upper GI bleeding who underwent CTa and DSA as the first diagnostic step followed by immediate coiling [8,20].

In summary, the clinical presentation of patients with upper GI bleeding can be diverse. The first step in patient management is to localize its cause with an emergency endoscopy in accordance with guidelines [4,9,19]. However, there are always exceptions to the rule. Patients can deteriorate rapidly, and it remains crucial to rely on the agreement of experts from different disciplines. We are fortunate to have a dedicated emergency intervention team that is always on standby. This team has the expertise to make quick decisions and change these decisions if necessary. Success can only be guaranteed if this team can rely on up-to-date facilities and technologies.

## 8 Conclusion

Guidelines for upper GI bleeding and life-threatening haemorrhagic shock mainly consider intraluminal pathologies. In these cases, an emergency endoscopy is recommended as the first step, possibly followed by an intervention. Obviously, there are no recommendations for exceptional presentations of upper GI bleeding. In those cases, the approach should be based on rapid decisions made by a multidisciplinary intervention team with access to hybrid multifunctional units.

## Patients’ perspective

I had severe pain on admission that got worse and was barely tolerable. The complete diagnostic analysis was performed by several medical specialists who together formed one team. They decided to perform angiography to block an artery. It took them less than half an hour to complete the procedure. My recovery is complete, and I am not experiencing any damage.

## Ethical approval

Not necessary.

## Sources of funding

There are no sources of funding

## Authors contribution

Donald Schweitzer, Sanne de Boer, Roel Bogie and Stefan Bouwense = Study concept, Data collection, and surgical therapy for the patient.

Donald Schweitzer, Dave Schweitzer and Stefan Bouwense = Writing - original draft preparation.

Donald Schweitzer, Sanne de Boer, Roel Bogie, Dave Schweitzer Stefan Bouwense, Daniel Keszthelyi = Editing and writing.

Donald Schweitzer, Stefan Bouwense = Senior author and manuscript reviewer.

## Consent

Written informed consent was obtained from the patient for publication of this case report and accompanying images. A copy of the written consent is available for review by the Editor-in-Chief of this journal on request.

## Registration of research studies

1. Name of the registry:

2. Unique Identifying number or registration ID:

3. Hyperlink to your specific registration (must be publicly accessible and will be checked):

## Guarantor

Donald Schweitzer and Stefan Bouwense.

## Provenance and peer review

Not commissioned, externally peer reviewed.

## Declaration of competing interest

There are no conflicts of interest regarding all authors.
